# Lichens and Health—Trends and Perspectives for the Study of Biodiversity in the Antarctic Ecosystem

**DOI:** 10.3390/jof11030198

**Published:** 2025-03-04

**Authors:** Tatiana Prado, Wim Maurits Sylvain Degrave, Gabriela Frois Duarte

**Affiliations:** 1 Laboratory of Applied Genomics and Bioinnovation, Oswaldo Cruz Institute, Oswaldo Cruz Foundation (FIOCRUZ), Av. Brasil, 4365, Manguinhos, Rio de Janeiro 21040-360, RJ, Brazil; wim.degrave@fiocruz.br (W.M.S.D.); gfroisduarte@gmail.com (G.F.D.); 2 Federal University of Rio de Janeiro (UFRJ), Av. Pedro Calmon, 550, Rio de Janeiro 21941-901, RJ, Brazil

**Keywords:** Antarctica, biotechnology, climate changes, environmental health, lichens, symbiosis

## Abstract

Lichens are an important vegetative component of the Antarctic terrestrial ecosystem and present a wide diversity. Recent advances in omics technologies have allowed for the identification of lichen microbiomes and the complex symbiotic relationships that contribute to their survival mechanisms under extreme conditions. The preservation of biodiversity and genetic resources is fundamental for the balance of ecosystems and for human and animal health. In order to assess the current knowledge on Antarctic lichens, we carried out a systematic review of the international applied research published between January 2019 and February 2024, using the PRISMA model (Preferred Reporting Items for Systematic Reviews and Meta-Analyses). Articles that included the descriptors “lichen” and “Antarctic” were gathered from the web, and a total of 110 and 614 publications were retrieved from PubMed and ScienceDirect, respectively. From those, 109 publications were selected and grouped according to their main research characteristics, namely, (i) biodiversity, ecology and conservation; (ii) biomonitoring and environmental health; (iii) biotechnology and metabolism; (iv) climate change; (v) evolution and taxonomy; (vi) reviews; and (vii) symbiosis. Several topics were related to the discovery of secondary metabolites with potential for treating neurodegenerative, cancer and metabolic diseases, besides compounds with antimicrobial activity. Survival mechanisms under extreme environmental conditions were also addressed in many studies, as well as research that explored the lichen-associated microbiome, its biodiversity, and its use in biomonitoring and climate change, and reviews. The main findings of these studies are discussed, as well as common themes and perspectives.

## 1. Introduction

Antarctica is the coldest continent on the planet, with average annual temperatures that can reach −10 °C on the Antarctic Peninsula and −50 °C in the interior of the continent. Precipitation and air relative humidity are low but can vary depending on proximity to coastal areas. The scarcity of organic matter, the low temperature and the presence of intense solar radiation are the main factors that limit life in Antarctica, but, unlike many species, lichens present high ecological plasticity and can tolerate extreme environmental conditions [1].

Traditionally, lichens are composite organisms with a mutualistic relationship between a heterotrophic mycobiont (i.e., a fungus) and an autotrophic photobiont (usually green algae and, in some cases, cyanobacteria), where this fungal lifestyle includes around 20% of all known fungal species [1]. Nevertheless, recent studies have verified that lichens are multi-symbionts, i.e., complex multi-species associations, including bacteria and other fungi, algae, and even yeasts and viruses [1,2,3,4,5,6,7]. These bipartite or multipartite symbionts are the main vegetative component of the Antarctic terrestrial ecosystem, widely distributed in the continent and islands [8], which can be explained by their immense capacity to adapt to extreme environmental conditions.

Most lichens are extremely tolerant to desiccation and low temperatures, surviving for months to years in a state of cryptobiosis, and to UV radiation, because many of their diverse secondary metabolites act as UV filters [9,10,11]. However, the mechanisms by which lichens tolerate extreme stress are still not completely understood and have been the subject of several studies, most of them suggesting that these adaptations rely on the physiological integration of the symbionts [10,11]. This physiological integration favors the adaptation of lichens in different types of environments, from arid regions of hot climate to alpine and polar regions, beyond tropical areas, and they are recognized to colonize a diversity of substrates, such as tree trunks, rocks and soil, among others [4]. Due to these characteristics, lichens can possibly be good models for the study of astrobiology [12].

Lichens can also be considered relevant species in the biomonitoring of atmospheric pollution, as they can accumulate trace elements or metals from the air and/or soil, thus acting as environmental health markers [4,13]. They also act as climate indicators, as some can tolerate large fluctuations in climate, while others require more specific regimes [14,15,16,17,18]. Lichens may be vulnerable to global warming, as the respiratory demand of the mycobiont partner may increase, requiring an increase in the proportion of cells of the photobiont partners to allow for survival and carbon balance [19].

Omics technologies have allowed for the expansion of knowledge about the molecular and taxonomic composition of different types of lichens, as well as gene expression patterns under different ecological conditions [4,13,20,21]. However, due to the complexity of these symbiotic systems and problems related to the formation of chimeric contigs, many studies are attempting the sequencing of individual partners cultivated axenically, although this approach cannot reveal the complete diversity of symbionts [7]. The virome of lichens may also contribute to multiple functions of the symbiotic system, although very little is known about viruses that infect components of these organisms [4].

Many secondary metabolites are produced by fungi, but other microorganisms that compose the symbiotic system are able to produce compounds with biotechnological value, and this characteristic makes the exploration of the lichen microbiome much more interesting [22,23]. The ability of lichen-forming fungi to produce antioxidant, anti-inflammatory and anticancer compounds and enzymes with diverse biotechnological applications has been investigated over the years, as has the ability of many symbiotic bacteria that make up lichens to produce bioactive compounds, especially with antimicrobial activity [24].

Given the global scenario of climate change and anthropogenic impact, emerging disease spread, and the need to develop new inputs for health, lichen research in the Antarctic ecosystem can present a promising approach both for environmental health and prospection for new molecular targets and biotechnological development in the field of health.

**Figure 1:**
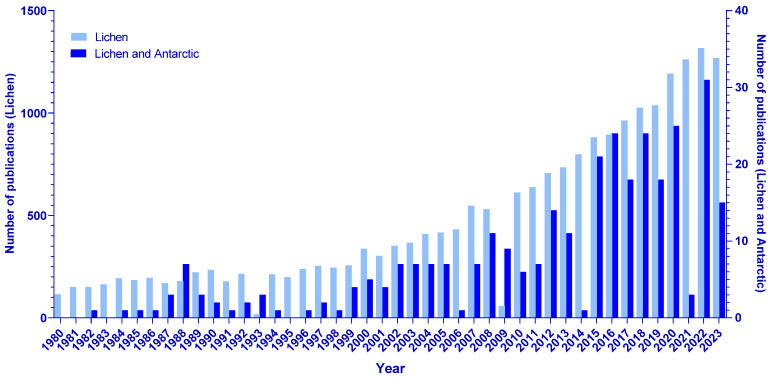
Number of publications in the PubMed database from January 1980 to December 2023 that include the descriptor “lichen” (light blue, with scale on the left); number of publications on lichens related to Antarctic ecosystems in the PubMed database from January 1980 to December 2023, including the descriptors “lichen” and “Antarctic” (dark blue, with scale on the right).

Research on lichens has increased considerably in the last 23 years, according to the PubMed database, and, following this trend, the same increase can be seen for research on lichens related to the Antarctic continent (Figure 1).

Antarctica is a continent with unique characteristics which has numerous natural resources that need to be preserved. Studies have reported more than 380 species of lichens on the Antarctic continent, with many new species recently described, including *Tephromela* Antarctica, *Umbilicaria* Antarctica and *Usnea* Antarctica, and at least 31 endemic species of the genera *Acarospora*, *Amandinea*, *Buellia*, *Leptogium*, *Pertusaria* and *Tetramellas* [25].

However, anthropogenic actions and global warming may affect this biodiversity, leading to ecosystem imbalances, with unpredictable impacts, such as increase, reduction or extinction of native species. Our review of lichen research in the Antarctic ecosystem over the past five years allowed us to identify trends and perspectives in the study of these organisms, as well as knowledge gaps that can be filled in the coming years, taking into account possible implications for the areas of clinical and environmental health.

## 2. Methods

**Figure 2:**
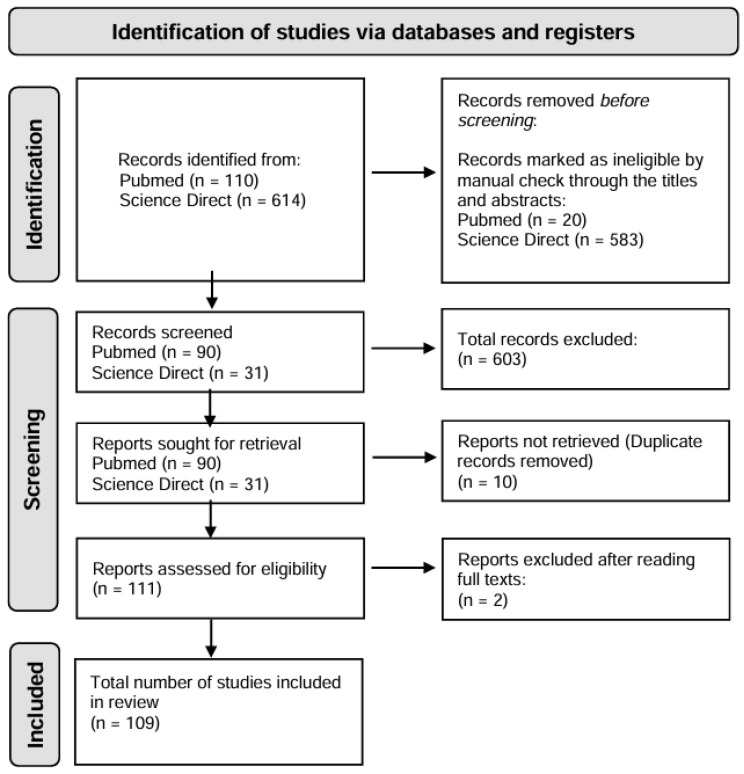
Flowchart of selection steps for studies related to lichen research in Antarctic ecosystem (January 2019 to February 2024).

The present study is a literature review based on the PRISMA (Preferred Reporting Items for Systematic Reviews and Meta-Analyses) model [26] (Figure 2). The PubMed and ScienceDirect databases were used to collect publications, using the descriptors “lichen” and “Antarctic”. The research was carried out considering the last 5 years (January 2019 up to February 2024).

This study included original research articles, short communications and reviews that addressed lichen research in the Antarctica ecosystem to evaluate the highlighted themes and research that have been prioritized by the conducting institutes, universities and government agencies, among others, in the last several years. Book and encyclopedia chapters, conference abstracts and news articles were not included in this review. Related articles that used meta-analysis data from the literature or discussed relationships between lichens and their symbiotic associations on the global scale in which the Antarctic continent was cited were also included in the analysis. For the selection of articles related to this area of study, the appearance of key terms was considered only in the fields of title and abstract. After reading the abstracts, articles that presented results or discussion about lichen research in Antarctica were included, thereby reducing the recovery of documents that contained the key terms casually but did not present any relation to the scope of this review. Duplicate articles, articles that were not related to the research theme and publications in other languages than English were also excluded from the study.

## 3. Results

The search in the databases retrieved 110 publications from PubMed and 614 from ScienceDirect, and, after reading the titles and abstracts, we selected 121 texts that addressed the topic more closely. After duplicate removal and full-text scrutiny, 109 publications were considered eligible for the preparation of the review, according to the flowchart in Figure 2.

**Figure 3:**
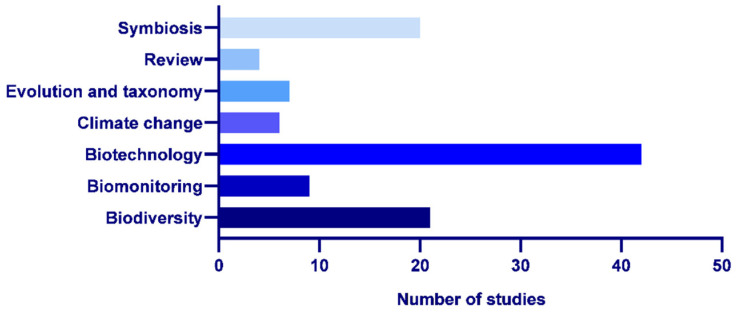
Number of studies included by thematic area (January 2019–February 2024).

The articles involving research on lichens in Antarctic ecosystems could be classified according to the main scope of the analysis, which we divided into the following themes: (i) biodiversity, ecology and conservation; (ii) biomonitoring and environmental health; (iii) biotechnology and metabolism; (iv) climate change; (v) evolution and taxonomy; (vi) reviews and (vii) symbiosis (Figure 3).

Biotechnology and metabolism (38.5%) was the area with a higher number of studies in the last several years, followed by biodiversity, ecology and conservation (19%); symbiosis (18.5%); biomonitoring and environmental health (8.5%); evolution and taxonomy (6.5%); climate change (5.5%); and reviews (3.5%) (Figure 3).

**Figure 4:**
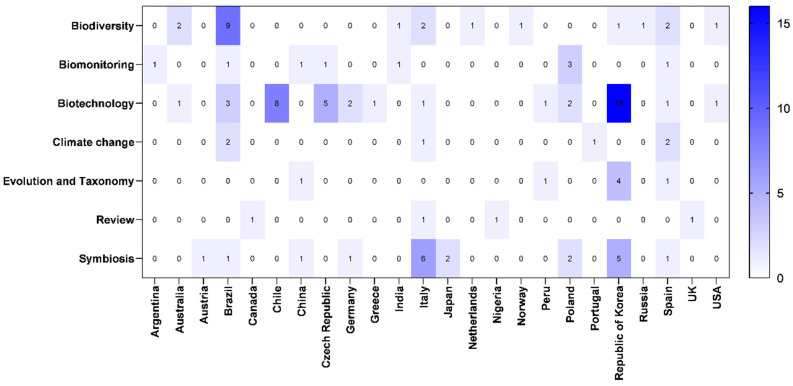
Heatmap showing the number of studies according to the country of the first author involved in lichen research in the Antarctic ecosystem by thematic area (January 2019–February 2024). Data spanned from white (low number of articles) to dark blue (higher number of articles), as illustrated by the color scale in the bar.

Figure 4 shows the distribution pattern, in percentages, of articles according to the country of origin of the first author.

The countries with the highest number of studies related to lichen research on the Antarctic continent published in the last five years included the Republic of Korea (23.8%), Brazil (14.5%), Italy (10%), Chile (7%), Spain (7%), Poland (6.5%) and the Czech Republic (5.5%). Other countries accounted for less than 3% of the total number of studies on this topic during this period (Figure 4). Many studies were carried out in collaborations involving different countries; therefore, some countries traditionally associated with research on the Antarctic continent [27] may not have appeared on this list (Figure 4). In any case, it is interesting to observe the interest of some South American countries in lichen research on the Antarctic continent (Figure 4).

A greater number of articles were related to research involving the potential of lichens in biotechnological applications. Research involving metabolomics and the description or discovery of secondary metabolites with potential antioxidant, anti-inflammatory, anticancer, cytotoxic or cytoprotective activities and protein inhibition too k the lead. Table 1 presents a list of the lichens, the secondary metabolites or bioactive compounds, and the potential biotechnological applications studied in these publications.

Some of the studies in the field of biotechnology and metabolism also involved research on metabolites in endolithic communities [3]. Mechanisms of activation and/or photoinhibition of photosynthesis under natural and laboratory conditions have also been explored in some eco-physiological studies using fluorescence and spectroscopy techniques [9,10,11,57,58,59,60,61,62].

Species composition, diversity and spatial coverage patterns were included in the thematic area of biodiversity, ecology and conservation [63,64,65,66,67,68,69]. The use of automated or semi-automated methods, such as semi-automatic object-based image analysis, remote sensing and satellite imagery, have been used to monitor the distribution patterns, productivity and biodiversity of lichens in Antarctica [68,70,71,72,73,74,75,76].

Articles addressing the theme of symbiosis involved the prospection of microbial communities (bacteria, yeasts, algae and fungi) that live in association with Antarctic lichens [16,52,54,56,77,78,79,80,81]. Many of these studies used metagenomic assays targeting 16S rRNA gene amplicon sequencing to explore the diversity of microbial communities, while others used whole- genome sequencing techniques to better understand their mechanisms of evolution [20,21]. Several studies aimed at characterizing these symbiotic microbial communities under different environmental conditions, considering the influence of different physicochemical parameters on their composition and functional profiles [2,5,6,7,15,16,48,50,52,80,82,83,84,85,86,87].

Studies using lichens as bioindicators of environmental contamination or in investigations into the sequestration of toxic compounds (e.g., novel brominated flame retardants), trace elements, metals and radionuclides were classified under the theme of biomonitoring and environmental health [88,89,90,91,92,93,94,95].

Although the topic of climate change is in the spotlight, few studies [15,17,18,96,97] could be classified in this area, although articles focusing on other themes sometimes included discussions of the future impact of their research on the issues of global warming and water scarcity. Perhaps the absence of consistent and verified data and the difficulty in carrying out long-term studies are reasons why studies directly related to climate change impacts on Antarctic biodiversity are scarce.

In evolution and taxonomy, studies involving population structure, gene flow, dating analyses, genealogical reconstruction methods, and phylogenetic analyses using molecular markers or target rDNA sequencing, aiming to establish the taxonomy of bacteria, fungi and algae symbionts in lichens, were observed [1,5,7,52,79,98].

A summary of the most frequently reported lichen species and their locations of occurrence can be consulted in Table 2.

Checklists on the diversity of lichen species already described on the Antarctic continent can be consulted in several references [16,25,65,99].

Finally, we observed a smaller number of reviews published in this period, covering topics such as biodiversity protection, survival mechanisms under extreme conditions and pharmacological potential [15,25,100,101].

## 4. Discussion

### 4.1. General Aspects

A trend of increasing interest in lichen research on a global scale and on the Antarctic continent was observed through a search for articles published in the last several years in databases that compiled relevant scientific productions from the selected period. The search for articles using the generic term “lichen” in the Pubmed database resulted in 19,431 items over the last 23 years (1980 to 2023), with a trend of expressive increase in research on the topic in the last 10 years. The same trend could be observed for lichen research on the Antarctic continent searched for using the descriptors “lichen” and “Antarctic”, which resulted in 315 articles published in the last 23 years, with a significant percentage made available in the last 10 years.

This review also showed that there is a tendency towards an increase in research related to the identification of metabolites with potential biotechnological applications. Due to the peculiar characteristics of the Antarctic continent, native or endemic species can represent potential sources for the discovery of new molecules, mainly applicable in the health sector.

No less important were studies on biodiversity conservation and long-term monitoring to assess the impact of anthropogenic activities on this biodiversity, including the global climate change scenario. The balance of ecosystems depends on maintaining Earth’s biodiversity, and this topic is also relevant to the researchers’ goals. Knowledge of lichens as symbiotic systems reveals that the diversity of associated microorganisms and their abundance can be variable depending on different abiotic gradients of the ecosystem. Therefore, it is necessary to study this microbial diversity in the face of the harsh climate and discover new microorganisms, genes and metabolic routes that, ultimately, could result in applied biotechnology research.

In this regard, it is not surprising that countries that are investing heavily in research and development and in the biotechnology industry, such as South Korea (the Republic of Korea) [102], are prominent in Antarctic research. There is also an expressive participation in research on lichens by developing countries in the South Atlantic and/or Latin American countries. Countries with greater geographic proximity to the Antarctic continent can be more directly affected by the consequences of environmental changes in this ecosystem, including disturbances caused by global warming, ecological imbalances, and the emergence or re-emergence of new or unknown pathogens. Advances in the technological capabilities of these countries to monitor the effects of anthropogenic impacts on Antarctic biodiversity may be boosting research in this field.

### 4.2. Secondary Metabolites and Potential Biotechnological Applications

Undoubtedly, lichens represent a rich source of bioactive molecules, which makes them interesting objects for the study of possible applications in the medical and biotechnological fields. In Antarctica, the potential for exploring new molecules or enzymes obtained from lichen extracts is promising given the unique characteristics of this ecosystem and the survival of several still unknown species.

Secondary metabolites of lichen have been shown to possess biological activities, such as antioxidant, analgesic and anti-inflammatory properties, amongst others [103]. Efforts toward the development of new drugs based on natural products are increasing worldwide [39,103,104], in part due to the emergence of antibiotic-resistant pathogens and new diseases, in addition to well-known diseases for which there are still no effective treatments, and the need to replace current medications due to toxicity or side effects associated with their use [39,104,105,106].

Lichen secondary metabolites have been widely studied using high-resolution chromatography (HPLC) techniques coupled to mass spectrometry (MS), and some studies have indicated that there are more than 1000 bioactive compounds currently registered [35,36,105]. The main metabolites studied in terms of biological activity include those derived from the acetyl– malonate pathway, such as depsides (e.g., atranorin), depsidones (e.g., salazinic acid) and dibenzofurans (e.g., usnic acid) [105,106]. The latter classes were shown to have anti-inflammatory activity and were able to inhibit the mRNA expression of inflammatory cytokines and mediators using a zebrafish larval model [28].

Antioxidant and cholinesterase inhibitory activities of these compounds have been demonstrated in recent metabolomic studies and may be promising for the treatment of diseases related to oxidative stress, such as Alzheimer’s, Parkinson’s and amyotrophic lateral sclerosis, and metabolic syndromes, such as diabetes mellitus [30,34,35].

Several other studies have shown the antioxidant activity and enzyme inhibitory potential of lichen ethanolic extracts, such as Areche et al.’s study [32] on *Himantormia lugubris* from Antarctica, in which the antioxidant activity and enzyme inhibitory potential (against cholinesterase and tyrosinase) of such extracts were observed. They detected 28 metabolites that showed enzymatic inhibitory activity. Since the inhibition of cholinesterase enzymes could play a role in the therapy of Alzheimer’s disease, these results illustrate the potential of this phenolic-enriched lichen to produce an extract with properties that can be used in neurodegenerative or related chronic nontransmittable diseases [32].

Ethanolic compounds present in extracts of *Cladonia chlorophaea* and *C. gracilis* species and their antioxidant activity and anti-inflammatory potential against pathologies such as asthma and other inflammatory disorders, such as allergic rhinitis and rheumatoid arthritis, have also been investigated [30]. Antarctic lichens, such as *Umbilicaria* Antarctica, *Cladonia cariosa* and *Himantormia lugubris*, can also produce compounds with inhibitory activity against tau protein, which is related to Alzheimer’s disease, in addition to other neurodegenerative disorders named tauopathies [31,41].

At present, there are many in vivo and in vitro experiments that have studied the anticancer effect of lichens [23,40,104]. Different species of lichens have demonstrated effects on lung cancer, prostate cancer, breast cancer, melanoma, colon cancer, liver cancer, leukemia, cervical cancer, rectal cancer, pancreatic cancer, ovarian cancer, lymphatic cancer, glioblastoma, astrocytoma and other cell lines [44,103]. The anticancer activities are attributed to cytotoxic action, cell cycle regulation, antiproliferation, anti-invasiveness, antimigration, antiangiogenesis, telomerase inhibition and inhibition of endothelial tube formation, among other pathways [39,104].

The genus *Usnea* also appears promising for the investigation of phenolic extracts and its potential antioxidant and antitumor activities, for example, its activity against melanoma [44]. Melanoma is a skin tumor and is related to excessive exposure of the skin to ultra-violet (UV) light. The UV radiation in Antarctica is very intense, and some organisms produce compounds that can absorb this radiation as a defense mechanism, as has been observed for several species of lichens that can produce relevant secondary metabolites that act in this protection [44].

Usnic acid, found in the genus *Usnea*, is one of the most abundant secondary metabolites with possible use in the treatment of various diseases, such as diarrhea, ulcers, urinary tract infections, tuberculosis and pneumonia, among others [44]. Vega-Bello et al. [44] identified usnic acid as the main metabolite present in the Antarctic lichen *Usnea aurantiaco-atra* and that the levels of this compound are much higher than those identified in other species of the same genus, such as *U. barbata*. High levels of this metabolite could be related to the environmental and climatic conditions in which lichens develop.

In addition to antioxidant, anti-inflammatory and anticancer activities, many substances produced by lichens, such as usnic acid, salazinic acid, evernic acid, protolichesterinic acid, isodivaricatic acid and divaricatinic acid, have been found to inhibit microbial pathogens, including bacteria, fungi and viruses [103]. Two new depsidones, himantormiones A and B (1 and 2), were isolated and identified from the Antarctic lichen *Himantormia lugubris* (Parmeliaceae) [33]. The isolated compounds were tested for antimicrobial and cytotoxic activities, and himantormione B (2) exhibited an inhibitory effect against *Staphylococcus aureus* [33]. Lee et al. [37] also discovered two new metabolic compounds isolated from *Ramalina terebrata*, collected from King George Island, Antarctica, in January 2021. The new compounds (stereocalpin B, a new cyclic depsipeptide, and a new dibenzofuran derivative) were tested for antimicrobial activities against *Escherichia coli*, *Staphylococcus aureus*, *Klebsiella pneumoniae*, *Candida albicans* and *Mycobacterium smegmatis*, but moderate antimicrobial activity was only observed against *E. coli* [37].

Other studies have demonstrated that a methanol– acetone extract—a crude extract from the Antarctic lichen *Usnea aurantiaco-atra*—shows antibacterial activity against *S. aureus* at a low concentration, and there is at least one bioactive compound that confers this activity [107]. This work also compared two species of lichens (*U. aurantiaco-atra* and *U.* Antarctica) and demonstrated that *U. aurantiaco-atra* presented greater cytotoxic and antibacterial activity than *U.* Antarctica, demonstrating the potential of this species for the isolation of antibacterial compounds in the Antarctic ecosystem [107].

Knowledge of the microbiome, genes and metabolic routes of symbiont microorganisms, such as yeasts and bacteria, has been explored in some studies [20,22,51,56,108], and potential applications of substances or enzymes have been highlighted for the detergent, food and biofuel industries and for the bioremediation of emerging contaminants.

Although lichen extracts have potential for the treatment of several diseases, some authors have pointed out the need to develop bioassay-guided fractionation and further isolation of minor compounds from crude extracts to accurately determine the bioactive compounds responsible for therapeutic activities that have been derived from these Antarctic species [32,44,51].

### 4.3. Physiological Properties and Survival in Extreme Environments

Unlike vascular plants, which have stomata or cuticles in their outer layers that help control water balance, lichens do not have the ability to regulate their water content [4]. Therefore, at first sight, one would assume that lichens would be quite sensitive to the extreme climatic conditions and low relative air humidity that are found in Antarctica. However, they have a poikilohydric lifestyle which enables them to survive extreme weather conditions for long periods in a dormant state [16]. In this review, we identified several studies that discuss survival and adaptation mechanisms of lichens in hostile environments, including resistance to alternating desiccation/rehydration cycles [9,10]. These investigations are particularly relevant for understanding the mechanisms and strategies for evolution and stress tolerance in conditions such as the absence of light, desiccation, low or high temperatures, and water scarcity. In lichens, physiological studies involving the photosynthetic apparatus and its protective mechanisms against extreme conditions have been reported [10,11].

Many of these studies verified the kinetics of hydration-induced activation of photosynthesis and often included analyse s of the activation or deactivation of photosynthesis under different conditions of light, water stress and hydration intervals at specific times [9,10,57]. Antarctic lichens, such as *Cladonia gracilis*, *C. borealis*, *Dermatocarpon polyphyllizum*, *Himantormia lugubris*, *Leptogium puberulum*, *Umbilicaria* Antarctica, *Usnea aurantiaco-atra* and *Xanthoria elegans*, have been tested in such experiments [9,10,11,57,60,62]. Most studies concluded that extremophile lichens have a mechanism to protect the chloroplastic apparatus of photobiont partners, especially when the water content is below 20% [9,10,60].

Resistance to dehydration is an advantageous strategy and could even be applicable in conditions outside Earth, such as in outer space, which, in addition to severe dehydration, is characterized by a vacuum and a broader spectrum of irradiation [58,109]. Therefore, researchers are also investigating the behavior of residual water in thallus through advanced techniques such as relaxometry, spectroscopy and sorption isotherm analysis, expanding research in the fields of biotechnology and astrobiology [12,109,110]. Some lichen species can produce large amounts of hydrogen, even after being exposed to extreme conditions, such as high doses of UVB radiation and very low or high temperatures (−196 °C and/or +70 °C, respectively) [12]. An international and interdisciplinary consortium (BIOMEX) has analyzed several groups of organisms, including *Buellia frigida*, an endemic Antarctic lichen, in experiments in outer space and under simulated space conditions [58,111]. Preliminary results demonstrated that a significative percentage of the tested lichens can survive in extreme conditions but a re sensitive to different doses of irradiation [58]. The lichen *B. frigida* also seems to be sensitive to outer space conditions, especially due to the DNA damage observed under these conditions [111]. Therefore, there is still a large gap in knowledge on this topic, and scientists need to better understand the adaptation and resistance mechanisms of different lichen species under adverse conditions in outer space.

### 4.4. Biodiversity and Environmental Health

In the field of biodiversity, ecology and conservation, some studies evaluated the changes in associated communities of mosses– lichens along a pedoenvironmental gradient, i.e., how species diversity (richness, species composition and beta diversity) are related to physical–chemical parameters and the organic matter and water content of soil. Some authors have also demonstrated that the distribution of species can be affected by the presence of penguin, elephant seal and seabird colonies because the se species can influence nitrogen concentrations and the circulation of pollutants in the Antarctic ecosystem [63,112,113,114,115]. Collections from large areas, taxonomic analysis and plant inventory data have also been used to monitor biodiversity [25,64,65,69], and analysis of photographs has been performed to calculate plant community coverage of sampled areas over time [70,74].

The use of automated or semi-automated methods is especially valuable in Antarctica, as access is limited and conditions are extreme. Furthermore, climate change can affect the Antarctic ecosystem, interfering with the coverage and distribution patterns of different species of lichens, mosses and other species of native fauna and flora. Therefore, it is essential to detect biodiversity trends to understand how changes may affect these communities. In this regard, several recent studies have used remote sensing and lichen spectra and/or satellite pictures to evaluate the surface reflectance profile patterns for Antarctic biological soil crusts (algae, lichens and mosses), generating vegetation cover distribution maps that can be monitored over time [67,68,70,71,72,73,75,76]. Due to the increasing anthropogenic impacts in the region, Phillips et al. [66] suggested that a greater number of Antarctic Specially Protected Areas (ASPAs) be adopted, considering the habitats and the patterns of occurrence and distributions of species as parameters for the establishment of protection areas [66].

The discovery of a variety of microorganisms that compose the symbiotic system of lichens and the characterization of the functional profiles of these communities was the third theme in evidence. Emphasis ha s been given to endolithic communities (found inside or embedded in rocks), a recent increase in the number of reports on which has been observed [2,3,6,15,79,81,85,86]. Lichen-forming fungal species were found in c ontinental Antarctica endoliths, revealing that these habitats may favor colonization by these symbiotic species in the most extreme climatic conditions [116,117]. The most abundant bacterial phyla identified from most of the lichen species analyzed in the studies were *Proteobacteria*, *Acidobacteria* and *Actinobacteria*, with variations in this abundance depending on the geographic characteristics of the areas and the substrates from which they were collected [2,47,52,82,85,117]. In summary, in most studies, the functional profiles of communities vary according to geographic regions and variable environmental conditions, such as altitude, spatial distance, sun exposure, temperature, humidity, soil organic matter content, deglaciation time and microclimatic conditions, in the case of lichen thallus parts [2,5,7,15,50,52,77,82,83,84,85,116,117]. Shrestha et al. [21] also identified CRISPR-Cas loci in the complete genomes of a lichen-associated *Burkholderia* sp. which may reveal different infection mechanisms in lichens. New Antarctic lichen-associated bacteria species have also been described [49,52,53].

In the area of biomonitoring and environmental health, the accumulation of radionuclides (^90^Sr, ^137^Cs, ^238,239+240^Pu and ^241^Am), selected natural radionuclides (^40^K, ^230,232^Th and ^234,238^U), trace elements (mainly Sm, La, Sc, Fe, Co, Hg and Ca), trace metals (Cd), and organic contaminants such as aliphatic hydrocarbons (e.g., petroleum and its by-products) in lichens (for example, *Usnea* Antarctica) has been addressed in some studies [89,90,91,92,93,94,95,97], and contamination levels have been found to vary according to whether locations are more or less impacted by human action, or influenced by glacier melt and penguin guano. In general, lichens are considered good biomarkers to evaluate the presence of these elements in the Antarctic ecosystem. Given the growing anthropogenic impact in the region from scientific, tourism and fishing activities, there is an increased expectation that lichens will be used in the biomonitoring of environmental health conditions.

Meta-analyses and field studies involving the identification of different species of lichens in specific localities and different climatic conditions have revealed that the macroclimate is the main driver of species distribution, making certain species useful as bioindicators of climatic conditions and, consequently, for assessing the consequences of climate change [2,14,16,17,18,19,118]. The Intergovernmental Panel on Climate Change (IPCC) has pointed to current global warming trends that could cause a rise in ocean levels, the loss of sea and land ice, and, consequently, negative impacts on global health and biodiversity [19,118]. Given the importance of this topic, especially considering the impact of climate change on Antarctic biodiversity and topics related to One Health (an approach that considers the integration between human, animal, plant and environmental health), it is expected that more studies will be conducted to characterize the diversity of lichens under climate change in this ecosystem.

### 4.5. Final Considerations

In recent years, research on lichens from the Antarctic continent has demonstrated that there is growing scientific interest in the discovery of new metabolites and their biotechnological applications, especially for human health and the production of new drugs for the treatment of infectious, metabolic, neurodegenerative and cancer diseases. As lichens are organisms formed by a symbiotic association involving multiple microorganisms, knowledge of the microbiome can help to elucidate distribution patterns and metabolic profiles, as well as the contribution of each partner to essential functions for survival in the most diverse and extreme environments on the planet.

The search for molecules and substances produced by lichens with bioactive properties is not new, but it has intensified in recent years because of technological advances and the interest in discovering new drugs that could, to some extent, be promising substitutes for synthetic drugs. Despite their potential, unique metabolites produced by lichens have received less attention by the pharmaceutical industry due to their slow growth and low biomass availability and the technical challenges involved in their artificial cultivation [119]. However, mass spectrometry-based metabolomics is a promising tool to detect and identify small molecules produced by the lichen microbiome and to understand the functional role of these microbial metabolites. The advancement of molecular engineering and synthetic biology could revolutionize the way medicines and other medical supplies are produced [119].

Often, secondary metabolites in extracts are explored through direct detection using HPLC and MS, but an alternative approach makes use of comparative genomics (“genome mining” strategies), which allows tracking of genes that encode proteins and families of genes and pathways that result in metabolites of interest [120]. The recent development of omics technologies linked to synthetic biology can assist in the expression of heterologous proteins using culturable hosts to provide specialized metabolites in a sustainable way [106,107,119]. This is particularly relevant, as the preservation of natural resources is imperative. Genetic engineering systems, when carefully controlled, can minimize the problem of overexploitation and extractivism and support the conservation of biological and genetic resources for future generations.

Environmental changes will have significant direct impacts on ecosystems. Considering the increase in the discharge of pollutants and the intensification of climate change, it is important to monitor and evaluate the effects of these changes on Antarctic biodiversity so that we have information that can guide the management and conservation of this territory, minimizing negative impacts on human, animal and environmental health.

## Figures and Tables

**Table 1:** Compounds with bioactive properties extracted from Antarctic lichens with potential biotechnological and health applications (data obtained from the literature: 2019–2024).

	Bioactive Compounds/Metabolite Type and/or Gene/Enzyme	Potential Application	References
**Lichen**			
*Amandinea* sp.	Crude extract	Anti-inflammatory effect	[28]
*Cladonia borealis* and *Stereocaulon alpinum*	Biosynthetic gene cluster (BGC)	Biosynthesis of atranorin	[29]
*Cladonia chlorophaea* and *Cladonia gracilis*	Usnic acid, thamnolic acid, 13D acid, perlatolic acid, psoromic acid, orsellinic acid, atranorin, cetraric acid, squamatic acid and methylorsellinate	Treatment of central nervous system pathologies, inflammatory disorders and metabolic alterations	[30]
*Cladonia cariosa* and *Himantormia lugubris*	Protolichesterinic acid, fumarprotocetraric acid and lichesterinic acid	Neurodegenerative disorders	[31]
*Himantormia lugubris*	Usnic acid, barbatolic acid, 5,7-dihydroxy-6-methylphthalide and atranol	Prevention of neurodegenerative or noncommunicable chronic diseases	[32]
*Himantormia lugubris*	Himantormione 2 (depsidone)	Inhibitory effect against *Staphylococcus aureus* and cytotoxic activity against HCT116 cells (colon cancer)	[33]
*Lecania brialmontii*, *Pseudephebe pubescens* and *Sphaerophorus globosus*	Barbatic acid, lecanoric acid, sphaerophorin and sekikaic acid	Potential inhibition of cholinesterase enzymes as a therapeutic target for neurodegenerative diseases (Parkinson’s and Alzheimer’s diseases)	[34]
*Placopsis contortuplicata*, *Ochrolechia frigida* and *Usnea* Antarctica	2,5DHA (2,5-dihydroxyterephthalic acid), cyperine, diospyrol, hypoxyphenone, lecanoric acid, orsellinic acid, prephenic acid, SDA (succinyldisalicylic acid) and (I) O4BBA (o-(4 biphenylylcarbonyl) benzoic acid	Treatment of metabolic diseases (diabetes mellitus) and those related to oxidative damage, such as Alzheimer’s, Parkinson’s and amyotrophic lateral sclerosis	[35]
*Pleurosticta acetabulum* (polyextremophilic behavior—not necessarily found in Antarctica)	Hydrogen after lichen exposure to extreme conditions	Promising fuel for the future and astrobiotechnological applications	[12]
*Ramalina terebrata*	Steroids, triterpenoid and an anthraquinone derivative	Chemotaxonomic marker	[36]
*Ramalina terebrata*	Cyclic depsipeptides (stereocalpin A/B), a new dibenzofuran derivative and 1,3,7,9-tetrahydroxy-2,8-dimethyl-4,6-di(ethanoyl)dibenzofuran	Antimicrobial, antiproliferative and anti-inflammatory	[37]
*Ramalina terebrata*	Ramalin	Alzheimer’s disease treatment	[38]
*Stereocaulon alpinum*	*p*-terphenyls	Cytotoxicity against HCT116 cells and anti-inflammatory activity	[39]
*Stereocaulon caespitosum*	Atranorin	Anticancer therapeutic—hepatocellular carcinoma (HCC)	[40]
*Umbilicaria* Antarctica	Tenuiorin	Treatment of Alzheimer’s disease	[41]
*Umbilicaria* Antarctica, *Sphaerophorus globosus*, *Coelopogon epiphorellum* and *Himantormia lugubris*	Lobaric acid, sphaerophorin, subsphaeric acid and barbatolic acid	Antioxidant activity	[42]
*Umbilicaria* Antarctica	Crude extract (mix of compounds)	Inflammatory diseases (rheumatoid arthritis, asthma and cancer)	[43]
*Usnea aurantiaco-atra*	Usnic acid	Cytotoxic activity (activity against some types of cancer, e.g., melanoma)	[44]
*Usnea aurantiaco-atra*	Dibenzofuran derivative, three phenolics, a p-terphenyl and three sterols	Chemotaxonomic marker	[45]
**Lichen-associated bacteria/fungi/yeasts**			
*Actinobacteria Streptacidiphilus carbonis*—lichen (*Sphaerophorus globosus*)-associated bacterium	Dipeptides and alkaloids	Antimicrobial effect	[23]
*Acremonium* sp. SF-7394 (fungus derived from unidentified Antarctic lichen)	Two new terpenoids: acrepseudoterin and isocordycepoloside A	Treatment of type 2 diabetes mellitus and obesity	[46]
Different taxa isolated (*Cetraria*, *Cladonia*, *Megaspora*, *Pseudephebe*, *Psoroma* and *Sphaerophorus*)—lichen-associated bacteria	Hydrolysis of macromolecules, such as skim milk, polymer and (hypo)xanthine; solubilization of inorganic phosphate; production of phytohormone indole-3-acetic acid; and fixation of atmospheric nitrogen	Hydrolysis of macromolecules/metabolism	[47]
*Hymenobacter* sp. PAMC 26554 and *Hymenobacter* sp. PAMC 26628—lichen-associated bacteria	Polysaccharide utilization-related carbohydrate-active enzyme	High percentage of hemicellulose degradation genes	[48]
*Hymenobacter* sp. PAMC 26554 isolated from Antarctic lichen (species not reported)	Citrate synthase	Metabolism	[49]
*Leptogium puberulum*-associated bacterial community	-	Catabolism of carbon compounds	[50]
*Lichenicola cladoniae* PAMC 26568—lichen *(Cladonia borealis)*-associated bacterium	β-1,4-D-glucan-degrading enzyme	Decomposition of cellulosic materials	[51]
*Lichenicola cladoniae* sp. nov. PAMC 26569—lichen (*Cladonia*)-associated bacterium	Genes for nitrogen fixation (nitrogenase, methane monooxygenases and methanol dehydrogenases)	Nitrogen metabolism (main function)	[52]
*Lichenihabitans psoromatis* sp. nov. PAMC 29148—lichen (*Psoroma* Antarcticum)-associated bacterium	4832 protein-coding genes	Metabolism of sugar and other organic compounds	[53]
*Pseudomonas*, *Caballeronia* and *Chryseobacterium* (main genera) isolated from *Usnea auratiaco-atra*, *Caloplaca regalis* and *Xanthoria candelaria* (main species)	Metabolic routes	Phosphate solubilization/agricultural sector (reduction in the use of chemical fertilizers)	[54]
*Rhodococcus strains* PAMC 28705 and PAMC 28707 isolated from Antarctic lichens (species not reported)	Carbohydrate-active enzymes	Phytopathogenic properties	[55]
*Shigella* sp. PAMC 28760—lichen (*Himantormia* sp.)-associated bacterium	91 genes related to carbohydrate-metabolizing enzymes	Carbohydrate metabolism	[20]
*Variovorax* sp. PAMC 26660—lichen-associated bacterium	Xenobiotic metabolism-related genes	Bioremediation	[22]
Yeast (several species) isolates from lichens: *Lecania brialmontii*, *Usnea aurantiaco-atra* and *Polycauliona candelaria* (main species)	Extracellular enzymes: protease, cellulase, esterase, amylase, lipase and pectinase	Metabolism of different sources of molecules, maintenance of symbiosis and survival of lichens under adverse conditions, and other biotechnological interests	[8]
Yeasts: *Vishniacozyma* and *Cystobasidium* (main genera)—lichen-associated yeasts (*Usnea auratiacoatra*, *Polycauliona regalis* and *Lecania brialmonti*) (main species)	Metabolic routes	Phosphate solubilization	[56]

**Table 2:** Lichen species observed in the Antarctic region (2019–2024). Species/location/reference.

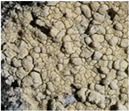 **[25]**	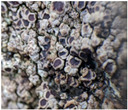 **[a]**	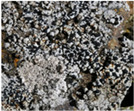 **[25]**	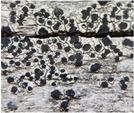 **[b]**	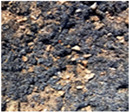 **[b]**	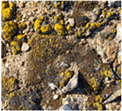 **[76]**
*Acarospora flavocordia*	*Acarospora macrocyclos*	*Amandinea latemarginata*	*Buellia augusta*	*Buellia pallida*	*Candelariella flava*
Maxwell Bay	Half Moon Island; South Shetland Islands	Maxwell Bay	Maxwell Bay	Grovnes Island	Osmar Island
[25]	[65]	[25]	[25]	[76]	[76]
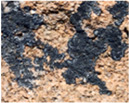 **[76]**	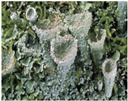 **[b]**	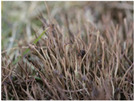 **[c]**	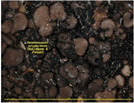 **[b]**	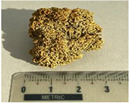 **[b]**	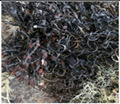 **[82]**
*Carbonea capsulate*	*Cladonia chlorophaea*	*Cladonia gracilis*	*Dermatocarpon polyphyllizum*	*Gondwania regalis*	*Himantormia lugubris*
McLeod Island	King George Islands	King George Island; Maxwell Bay	Shetland Islands	King George Island	King George Island; South Shetland Islands
[76]	[35]	[25,35,62]	[9]	[82]	[7,32,60,62,82]
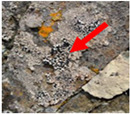 **[8]**	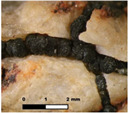 **[16]**	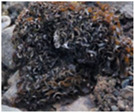 **[25]**	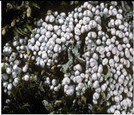 **[b]**	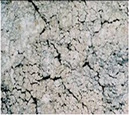 **[c]**	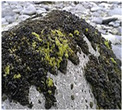 **[c]**
*Lecania brialmontii*	*Lecidea cancriformis*	*Leptogium puberulum*	*Lepra dactylina* (newly reported species)	*Lepraria* sp.	*Mastodia tessellata*
South Shetland Islands	Southwest Ross Sea coast	King George Island	Maxwell Bay	Osmar Island	Barrientos Island
[8,34,56]	[16]	[9,25,82]	[25]	[76]	[8]
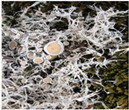 **[b]**	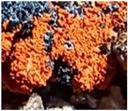 **[76]**	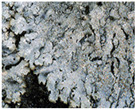 **[b]**	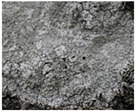 **[25]**	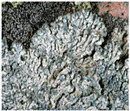 **[b]**	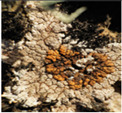 **[7]**
*Ochrolechia frigida*	*Oxneria huculica*	*Parmelia saxatilis*	*Pertusaria signyae*	*Physcia caesia*	*Placopsis* Antarctica
King George Island	Osmar Island	King George Island	Maxwell Bay	Fisher Island	King George Island; South Shetland Islands
[35]	[76]	[60]	[25]	[76]	[7,60]
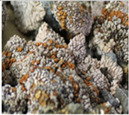 **[76]**	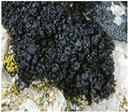 **[b]**	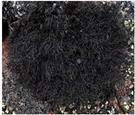 **[b]**	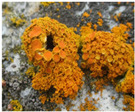 **[b]**	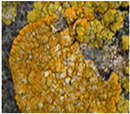 **[8]**	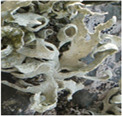 **[82]**
*Placopsis contortuplicata*	*Pseudephebe minuscula*	*Pseudephebe pubescens*	*Polycauliona candelaria*	*Polycauliona regalis*	*Ramalina terebrata*
King George Island	Grovnes Island	South Shetland Islands	Antarctic Peninsula	Barrientos Island	King George Island
[35,76]	[76]	[34]	[8]	[8]	[7,36,82]
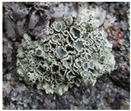 **[b]**	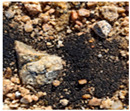 **[76]**	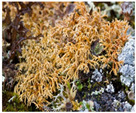 **[b]**	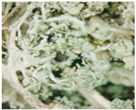 **[25]**	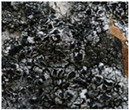 **[25]**	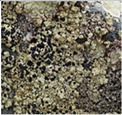 **[25]**
*Rhizoplaca melanophthalma*	*Shackletonia siphonospora*	*Sphaerophorus globosus*	*Stereocaulon caespitosum* (newly reported species)	*Tephromela* Antarctica	*Tetramelas anisomerus*
Easter Island	Brokness Island	Snow Island; South Shetland Islands	Maxwell Bay	Maxwell Bay	Maxwell Bay
[76]	[76]	[8,34]	[25]	[25]	[25]
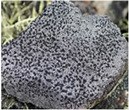 **[25]**	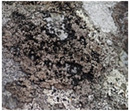 **[25]**	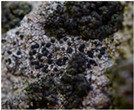 **[25]**	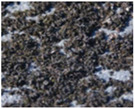 **[82]**	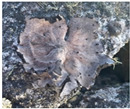 **[d]**	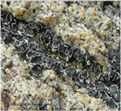 **[e]**
*Tetramelas darbishirei*	*Tetramelas grimmiae*	*Tetramelas granulosus*	*Turgidosculum complicatulum*	*Umbilicaria* Antarctica	*Umbilicaria aprina*
Maxwell Bay	Maxwell Bay	Maxwell Bay	King George Island	Shetland Islands	Brokness Island
[25]	[25]	[25]	[82]	[9,25,35,41]	[76]
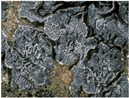 **[b]**	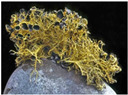 **[b]**	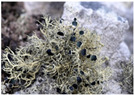 **[a]**	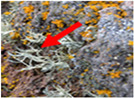 **[8]**	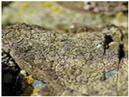 **[25]**	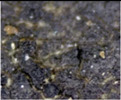 **[25]**
*Umbilicaria decussata*	*Usnea* Antarctica	*Usnea aurantiaca-atra*	*Usnea capilacea* (red arrow)	*Verrucaria psychrophila*	*Wahlenbergiella striatula* (newly reported species)
Cecilia Island; Fisher Island	King George Island	King George Island	Deception Island	Maxwell Bay	Maxwell Bay
[8,76]	[82]	[8,44,62,82,88]	[8]	[25]	[25]

Photos of the lichens were obtained from the cited articles or the following sites: [a] iNaturalist Guatemala; [b] Consortium of Lichen Herbaria, 2024 (lichenportal.org); [c] Wikipedia; [d] BioDiversity4All; [e] Antarctica NZ. All the material included in this table is licensed under a Creative Commons International License.

## Data Availability

Data obtained for lichen figures can be consulted through the following websites: https://guatemala.inaturalist.org/; the Consortium of Lichen Herbaria (lichenportal.org); Wikipedia3; BioDiversity4All4 (https://www.biodiversity4all.org/login); Antarctica NZ5 (https://www.antarcticanz.govt.nz/). Accessed on 1 March 2024. All the material included in this article is licensed under a Creative Commons International License.
